# Effect of climatic variability on childhood diarrhea and its high risk periods in northwestern parts of Ethiopia

**DOI:** 10.1371/journal.pone.0186933

**Published:** 2017-10-26

**Authors:** Muluken Azage, Abera Kumie, Alemayehu Worku, Amvrossios C. Bagtzoglou, Emmanouil Anagnostou

**Affiliations:** 1 School of Public Health, College of Medicine and Health Sciences, Bahir Dar University, Bahir Dar, Ethiopia; 2 Ethiopian Institute of Water Resources, Addis Ababa University, Addis Ababa, Ethiopia; 3 School of Public Health, College of Health Sciences, Addis Ababa University, Addis Ababa, Ethiopia; 4 Department of Civil and Environmental Engineering, School of Engineering, University of Connecticut, Storrs, CT, United States of America; Columbia University, UNITED STATES

## Abstract

**Background:**

Increasing climate variability as a result of climate change will be one of the public health challenges to control infectious diseases in the future, particularly in sub-Saharan Africa including Ethiopia.

**Objective:**

To investigate the effect of climate variability on childhood diarrhea (CDD) and identify high risk periods of diarrheal diseases.

**Methods:**

The study was conducted in all districts located in three Zones (Awi, West and East Gojjam) of Amhara Region in northwestern parts of Ethiopia. Monthly CDD cases for 24 months (from July 2013 to June 2015) reported to each district health office from the routine surveillance system were used for the study. Temperature, rainfall and humidity data for each district were extracted from satellite precipitation estimates and global atmospheric reanalysis. The space-time permutation scan statistic was used to identify high risk periods of CDD. A negative binomial regression was used to investigate the relationship between cases of CDD and climate variables. Statistical analyses were conducted using SaTScan program and StataSE v. 12.

**Results:**

The monthly average incidence rate of CDD was 11.4 per 1000 (95%CI 10.8–12.0) with significant variation between males [12.5 per 1000 (95%CI 11.9 to 13.2)] and females [10.2 per 1000 (95%CI 9.6 to 10.8)]. The space-time permutation scan statistic identified the most likely high risk period of CDD between March and June 2014 located in Huletej Enese district of East Gojjam Zone. Monthly average temperature and monthly average rainfall were positively associated with the rate of CDD, whereas the relative humidity was negatively associated with the rate of CDD.

**Conclusions:**

This study found that the most likely high risk period is in the beginning of the dry season. Climatic factors have an association with the occurrence of CDD. Therefore, CDD prevention and control strategy should consider local weather variations to improve programs on CDD.

## Introduction

Climate change due to anthropogenic and natural activities has been observed at global levels and will result in increased climate variability in different geographical locations [1]. Climate change refers to the long-term significant change in the “average weather” that a given region experiences, while climate variability refers to variation in the mean state and other statistics of climate on all temporal and spatial scales beyond that of individual weather events [2].

In sub-Saharan Africa, and Ethiopia in particular, the projected climate scenarios show an increase in rainfall variability and temperature as well as prolonged droughts in the region [3]. The expected changes in climate will result in shifts in the spatiotemporal variation of diseases and increase the occurrence of infectious disease outbreaks [2]. The World Health Organization (WHO) also reported that climate variability (temperature, rainfall and relative humidity) will be one of the public health challenges to control infectious diseases in the future, particularly in low-income developing countries [4].

Studies found scientific evidence on the link between climate variability and infectious diseases [5, 6]. Diarrhea, one of the infectious diseases, was also associated with climate variability [4]. Studies have shown that climate factors significantly affect seasonal diarrhea in susceptible populations, particularly children under five years of age [7, 8]. Common bacterial pathogens associated with diarrhea (eg. diarrheagenic Escherichia coli and others) replicate rapidly in increased ambient temperature. Moreover, high temperature also extends the survival of these pathogens in the external environment [9–11]. Viral pathogens that cause diarrhea such as rotavirus are more dominant in lower ambient temperature and the cooler periods extend the survival of this pathogens [12]. Excess cases of diarrhea have been reported after heavy rainfall, and associated extreme hydrologic conditions like floods and drought [4, 13]. For example, flooding and surface runoff increase the rate of contamination of drinking water sources at the beginning of the rainy season [14, 15]. Diarrheal diseases are linked to droughts due to water scarcity and the high burden of malnutrition [16, 17].

Studies conducted in Ethiopia revealed that increased average temperature, increased frequency of extreme weather conditions and shifting the beginning period of rain either early or late were observed [18, 19]. Droughts and floods are also very common and some of these have been associated with El Niño and the southern oscillation (ENSO) events in Ethiopia [20]. Such climatic variability and extreme weather conditions trigger the occurrence of diarrhea morbidity. Increases in the frequency and severity of floods and droughts are expected to exacerbate the occurrence of diarrheal diseases due to deterioration in water quality, water scarcity and increasing burden of malnutrition [17].

The burden of childhood diarrhea is high in Ethiopia and diarrhea was the second leading cause of childhood deaths in the country [21]. Diarrhea was also one of the top five causes of child morbidity in the past five years in Amhara Region where the study area is located [22–26]. However, studies on the effect of climate variability on diarrhea and its high risk period have not been conducted in the region, particularly the northwestern parts of Ethiopia. Predicting the incidence using time series data and identifying peak periods of disease have been used so far by public health officials for early detection of irregularity of the disease incidence and to act as an epidemic alert system [27–31]. However, in the absence of credible evidence of such information, it is difficult to draw effective disease prevention intervention strategies and to take timely countermeasures against the disease. Therefore, the aim of this study is to investigate the relationship between climate variability and childhood diarrhea, seasonality of diarrhea and climate variability, and identify high risk periods of diarrheal diseases for the northwestern parts of Ethiopia.

## Methods and materials

Facility based and meteorology data were linked to address the objectives of the study. Retrospective data on children under-five years of age with diarrhea were extracted from the Health Management Information System at the district level reported monthly to the routine surveillance system of the Zonal health office over a two year period (24 months) from July 1, 2013 to June 30, 2015. Meteorological data were obtained from satellite precipitation data and global atmospheric reanalysis data.

### Study area

**Fig 1:**
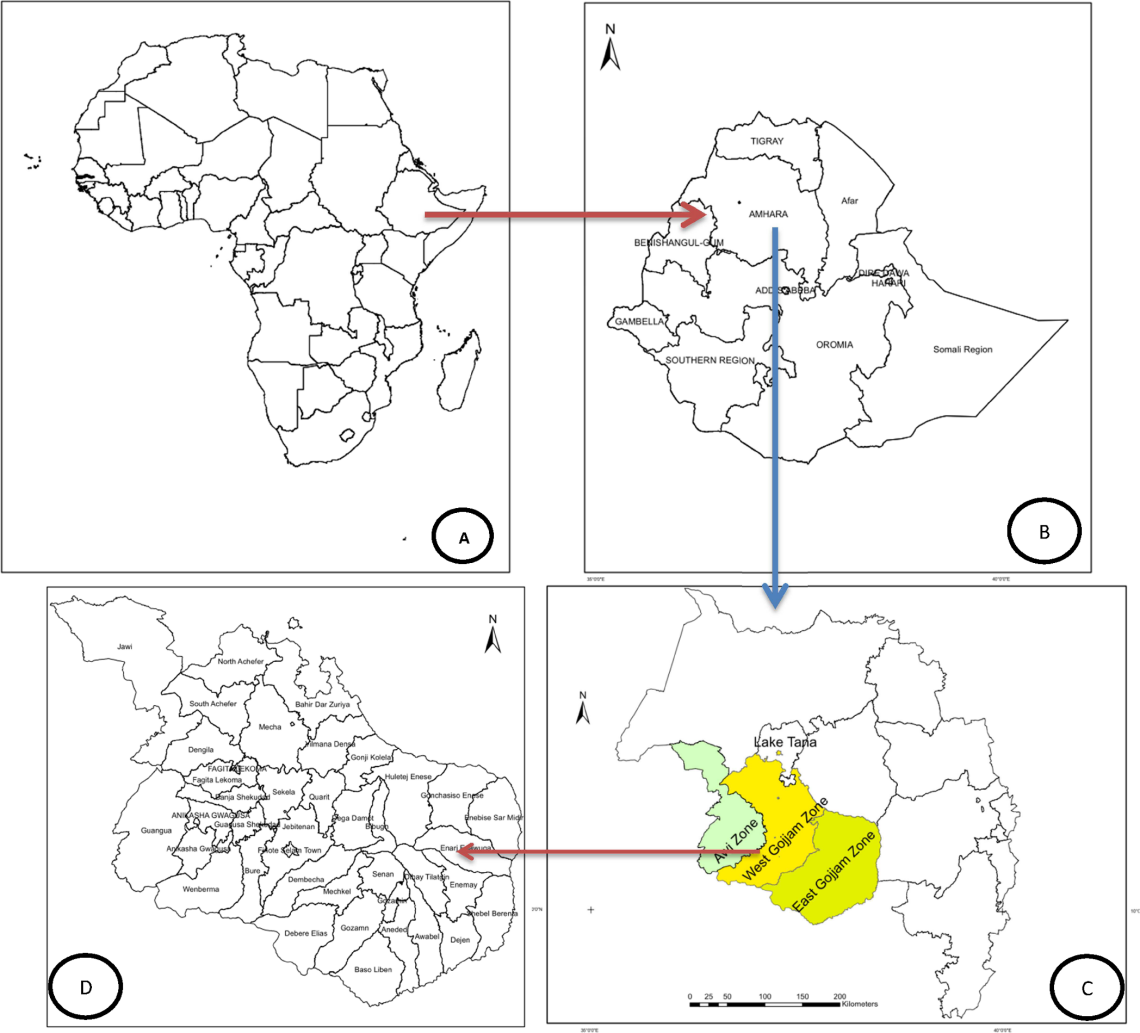
Map of Africa (A), Ethiopia (B), Amhara Region (C) and districts in the study area (D).

The study was conducted in all districts located in three Zones of Amhara National Regional State (Awi, West and East Gojjam Zones) in northwestern parts of Ethiopia (Fig 1). Based on the 2007 National Population and Housing Census of Ethiopia, the projected population for 2013/14 in the study area is about 6,102,870, of which 2,998,149 (49.1%) were male, 826,246 (13.5%) lived in urban areas, and 708,430 (11.6%) were children under five years of age [32]. Primary health care services such as disease promotion, prevention and control are given in the study area. Health care services given at health centers and health posts are reported to the district health office monthly. Three seasons, namely, dry season from October to February, pre-rainy season from March to May followed by a long rainy season from June to September, occur in the study area. The mean temperature per month in the study districts varies in the range between 14.5°C and 34.5 ^o^C during dry season. The rainfall and relative humidity per month in the study districts are in the range from 0 mm to 77 mm and from 33 to 73.4, respectively, during dry season. During the main rainy season, the monthly rainfall in the study areas ranges from 158 mm to 428 mm and the average monthly temperature ranges from 14.5 ^o^C to 20.6 ^o^C. During the short rainy season, the monthly rainfall in the study areas ranges from 45 mm to 144 mm and the average monthly temperature in the study districts varies in the range from 17.5 ^o^C to 22.4 ^o^C. The mean daily maximum and minimum temperatures in the study area are 26°C and 11°C, respectively [33].

### Data sources, measurement and collection

The Health Management Information System (HMIS) was established to report infectious diseases and other health care related services from health facilities to the Ethiopian Ministry of Health as a routine surveillance system since 2010. Data from all districts are being reported electronically to Health Management Information System Center at the Ministry of Health monthly since July 2013. Diarrhea is one of the reportable infectious diseases. A case of diarrhea in health facilities is recorded based on the WHO definition, which is if a person had three or more loose stools or watery diarrhea in a day. Data for children under five years of age with diarrhea were extracted from the Health Management Information System using the extracted format sheet. For this study, monthly childhood diarrhea cases from July 2013 to June 2015 were included during data analysis.

The projected population data of under-five children based on the results of the 2007 National Population and Housing Census of Ethiopia in a district for each year were obtained from the Amhara Regional State, Bureau of Finance and Economic Development. The projected population data of under-five children were used as the known underlying population at risk to calculate the monthly incidence rate of childhood diarrhea.

### Climate variability

**Fig 2:**
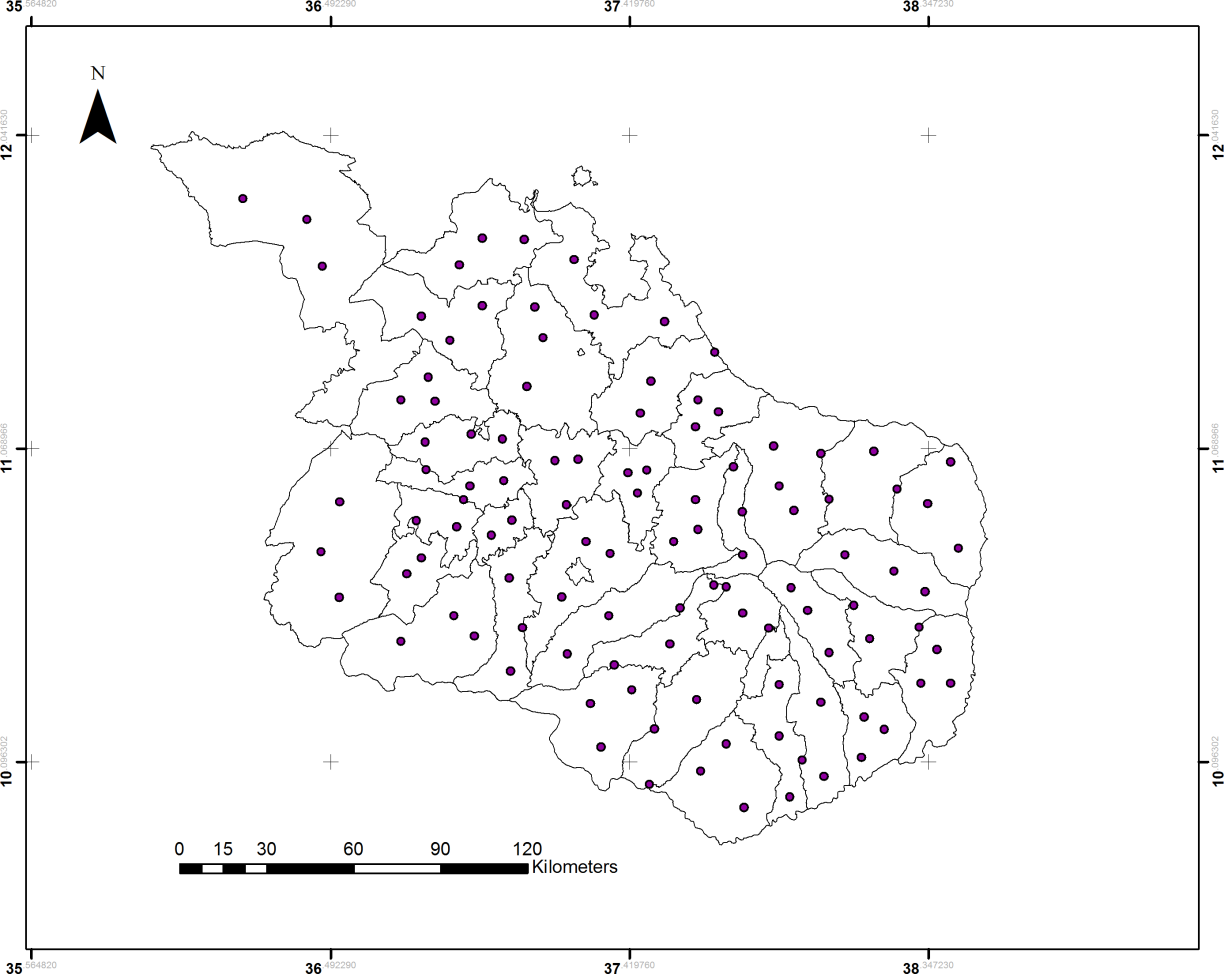
Points of longitude and latitude locations in each district used to extract climatic variables from satellite dataset.

Data of climate variability (rainfall, temperature and relative humidity) were obtained from Satellite data since each district with different locations in the study area had no meteorological data. Monthly average rainfall was extracted from the National Oceanic and Atmospheric Administration (NOAA) Climate Prediction Center morphing technique (CMORPH) [34] which includes gauge adjustment [35], while monthly average temperature, and specific humidity for each district in the period between 2013 July to 2015 June were extracted from the Global Land Data Assimilation (GLDAS) reanalysis system of NASA’s Goddard Space Flight Center (GSFC) [36]. Three geographical points in each district were chosen at different altitudes to represent each district in terms of climate variability. The geographical point of each location (latitude and longitude) was used to extract climatic data from the satellite and reanalysis datasets (Fig 2). Specific humidity was changed into relative humidity by using altitude, air pressure and the average temperature of each latitude and longitude location. The average monthly value of three locations of each climatic variable was calculated and used to investigate the relationship with monthly diarrhea cases. Climatic variability data obtained from satellite datasets were validated and published by other research groups elsewhere [35, 36]. Specifically, our research group evaluated nine satellite precipitation products over nine complex terrain regions around the globe, and a 13 year period of ground validation data, over the Blue Nile area [37]. Based on this evaluation CMORPH was shown to outperform all products. This is also consistent to findings by earlier studies [38–40] and is attributed to the fact that IR-based rainfall algorithms have limitations over mountainous regions of East Africa, while PMW is more physically based and free of cold surface effects in this sub-Tropical area.

### Data analysis

Descriptive statistics such as mean, standard deviation (SD) and range were used to describe climatic variability and the average incidence rate with 95% confidence interval (CI) was calculated to observe childhood diarrhea variation between males and females and also to show seasonal variability of childhood diarrhea.

### For high risk periods

The space-time permutation scan statistic was used to identify high risk periods of childhood diarrhea. The space-time permutation scan statistic is the recommended tool for early disease detection in any local and national diseases surveillance systems [41]. The space-time permutation scan statistic is able to detect significant high risk period of the disease by adjusting for purely spatial and purely temporal variations [42]. This model requires only case data with information about the spatial location and time for each case, with no need for population-at-risk data [41]. The space-time permutation scan statistic uses thousands or millions of overlapping cylindrical windows to scan the study area. The base of the cylinder represents space, and the height represents time. Both the circular geographic base and the start date, which are mutually independent, are flexible during scanning of the study area. The likelihood ratio test statistic is constructed using a computational algorithm to calculate the likelihood of each window. The spatial-temporal scan was used to test all possible circles for the bottom of the cylinder and all possible time periods for the height, each being a possible candidate for a high risk period. The area of the bottom circle was varied from zero to the maximum specified cluster size of the total cases. The height of the cylinder ranged from a single time period to a defined maximum number of time periods. In the study, minimum temporal cluster size was set at one month and maximum temporal cluster size was four months since the minimum period in the dataset is one month and the maximum temporal cluster size is set at four months since dry and rainy periods in the study area extend up to four months. The maximum spatial cluster size was performed with a default maximum spatial cluster size of 50% of the population. This fifty percent was specified as the upper limit which allowed both small and large clusters to be detected and ignored clusters that contain more than 50% of the population. The Poisson generalized likelihood ratio (GLR) was calculated for each window of observed and expected number of cases in order to identify statistically significant high risk periods. Among all of the cylinders evaluated, the one with the maximum GLR was the space-time cluster of cases that is the primary candidate for a true high risk period. The value of GLR is expressed as

$$GLR={\left(\frac{c_{A}}{\mu_{A}}\right)}^{c_{A}}\;{\left(\frac{C-c_{A}}{C-\mu_{A}}\right)}^{C-c_{A}}$$

where c_A_ and μ_A_ were the observed and expected number of cases in the cylinder, respectively and C was the total number of observed cases. A p-value for the scan statistic was calculated using Monte Carlo simulation from 999 simulated datasets [42], by comparing the rank of the maximum GLR from the real dataset and simulated datasets. A significance level of alpha < 0.05 was used to test whether a high risk period was significant.

#### For effect of climate variability

Correlation using spearman's correlation coefficient, bivariate and multivariable negative binomial regressions were used to examine the relationship between climatic factors and childhood diarrhea (outcome variable). The reason for using non-parametric statistics is the overdispersion of childhood diarrhea (data of childhood diarrhea is not normally distributed). Correlation analysis using spearman's correlation coefficient between childhood diarrhea and lag effect of climatic factors (lag zero, one and two) was performed to gain insights into the crude associations between climatic factors and the outcome variable [7]. A negative binomial time-series regression was also used to predict the incidence of childhood diarrhea using climate variables. A negative binomial (NB) regression accounts for overdispersion by adding an additional dispersion (variance) parameter to the Poisson model and this regression model can accommodate increased variability [43]. A bivariate negative binomial regression was performed to examine the crude associations between the climatic factors and the outcome variable. Multivariable negative binomial regression analysis was used to control the confounding effect between climatic factors. The multicollinearity between climate variables using the Variance Inflation Factor (VIF) was checked before the multivariable regression analysis. In multivariable regression, the VIF is used as an indicator of multicollinearity. Various recommendations for acceptable levels of VIF have been published in the literature, and range from a value of 10 [44–46] to 5 [47, 48]. In this study, a value of four and below for the VIF was used to control multicollinearity between climate variables at lag zero, one and two. In the multivariable negative binomial regression model, adjusted Incidence Rate Ratio (IRR) with 95%confidence interval was calculated to identify the independent effect of each explanatory variable with the outcome variable. A significance level of 0.05 was considered for all statistical tests.

Statistical analyses were conducted using SaTScan program [49] and StataSE v. 12 (StataCorp LP, USA). Specifically, SaTScan was used to carry out space-time permutation scan statistic to identify high risk periods of childhood diarrhea and StataSE v. 12 was used to describe data and conduct negative binomial regression analysis.

### Ethics approval

This research was conducted after having ethical approval by the research ethics committee of College of Medicine and Health Sciences, Bahir Dar University. The committee provided ethical approval after reviewing all components of the research protocol. Permission letters from the Amhara Regional Health Bureau and district health offices were also obtained before starting data collection. All the information was kept confidential, and no individual identifiers were collected during the retrieval of retrospective data.

## Results

### Epidemiologic characteristics

**Fig 3:**
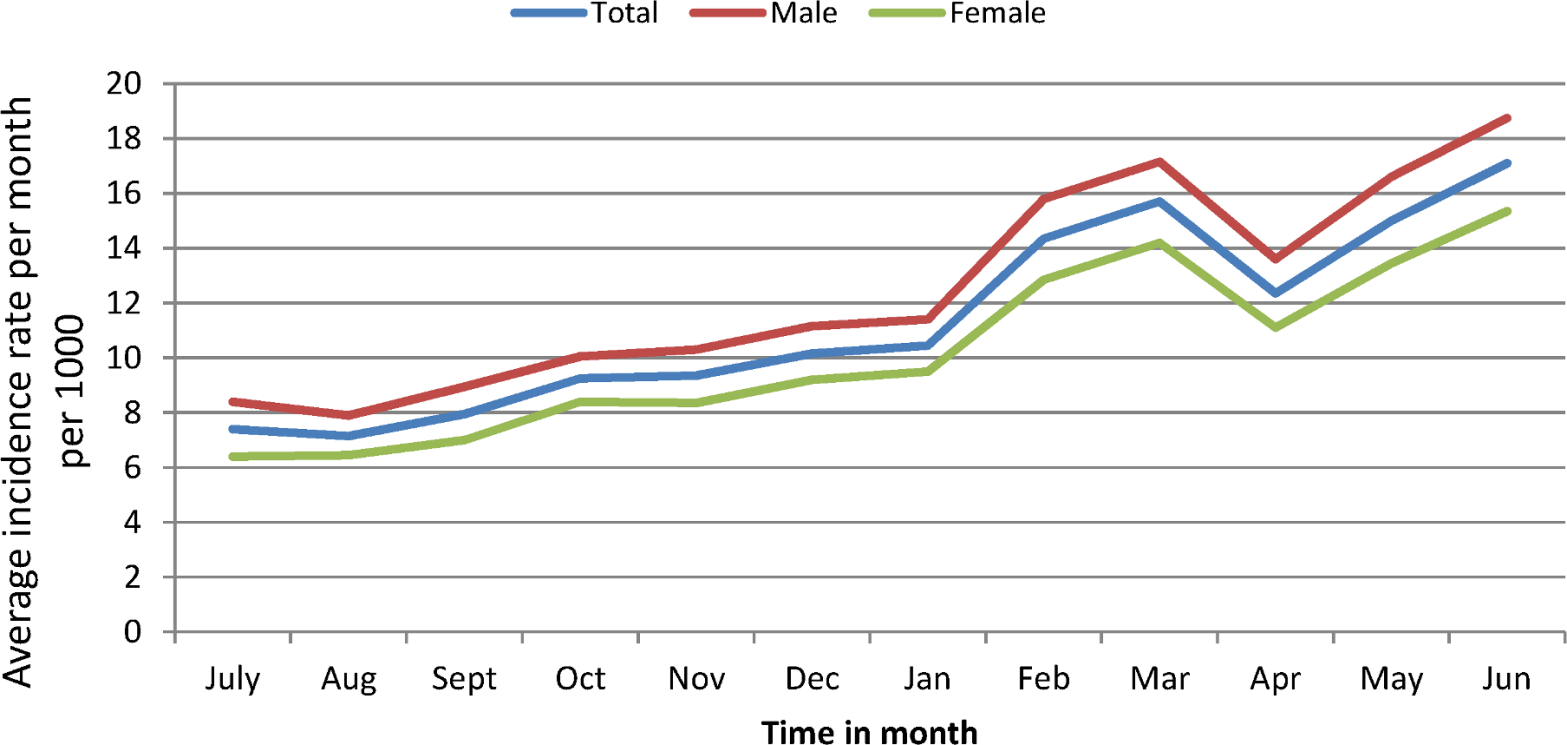
The average incidence rate per 1000 of each month across one year among males and females in northwest Ethiopia, from July 2013 to June 2015.

There were 217,734 cases of childhood diarrhea, of which 120,752 (55.5%) were males and 96,982 (44.5%) were females from July 1, 2013 to June 30, 2015. The monthly average incidence rate of childhood diarrhea was 11.4 per 1000 (95%CI 10.8–12.0) with significant variation between males [12.5 per 1000 (95%CI 11.9 to 13.2)] and females [10.2 per 1000 (95%CI 9.6 to 10.8)]. The incidence rate began to increase (above the annual average) in February (14.4 per 1000) and reached a peak rate of 17.1 per 1000 in June (18.8 per 1000 in males and 15.4 per 1000 in females). The lowest incidence rate was observed in July (7.4 per 1000) (Fig 3).

**Fig 4:**
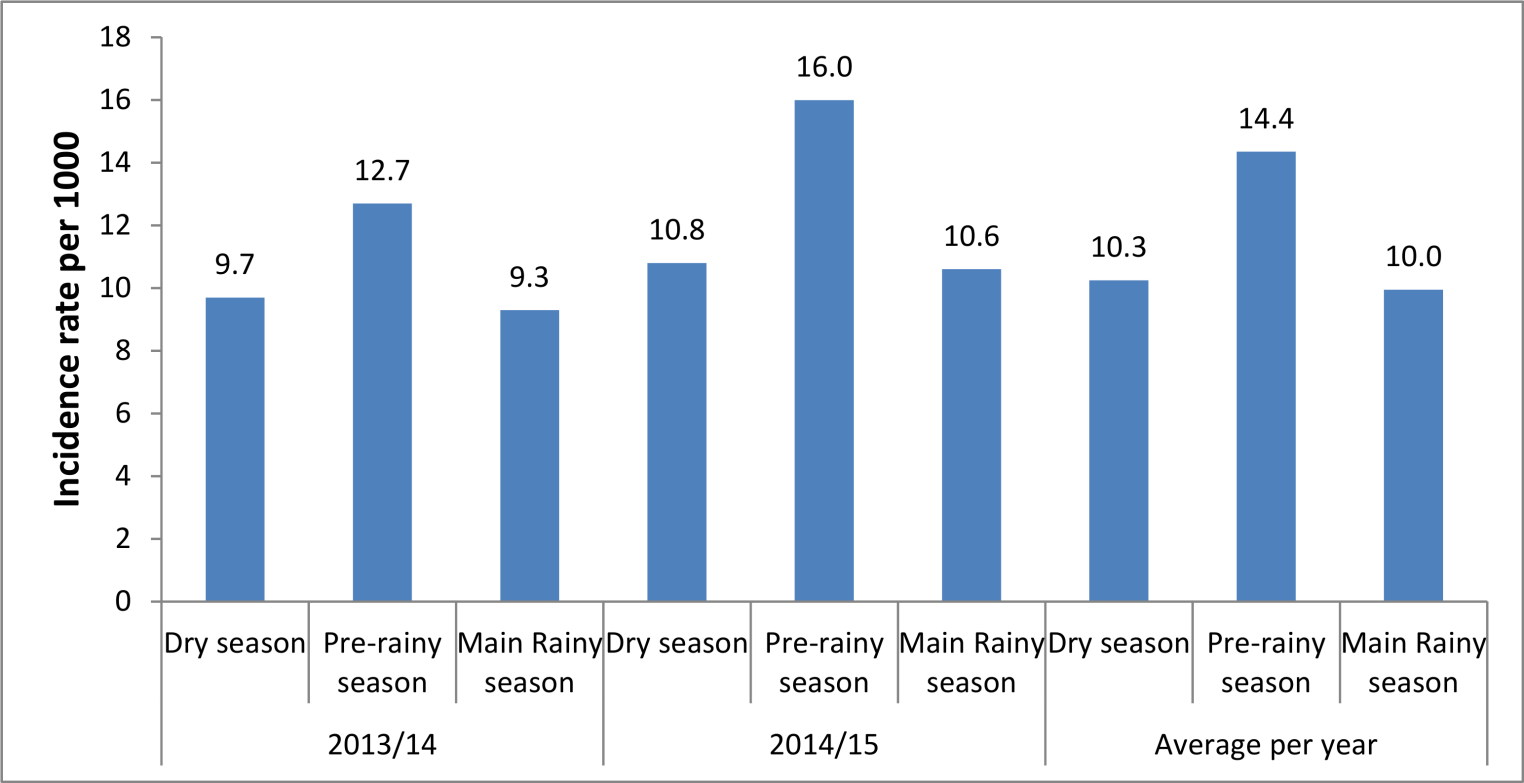
Monthly incidence rate per 1000 in dry, pre-rainy and main rain seasons in northwest Ethiopia, from July 2013 to June 2015.

Regarding the seasonal variation, the highest average incidence rate was observed during the pre-rainy season (March to May) (14.4 per 1000 per month with 95%CI of 13.0 to 15.6) whereas the lowest rate was observed in the main rainy season from June to September (10.0 per 1000 per month with 95%CI of 8.9 to 11.0). The average incidence rate from October to February (Dry season) was 10.3 per 1000 per month with 95%CI of 9.3 to 11.3 (Fig 4).

### High risk periods

The space-time permutation scan statistic identified high risk periods of childhood diarrhea with locations in the study area. The most likely high risk period was between March and June 2014 located in the Huletej Enese district of East Gojjam Zone. The center of the high risk period was at 11.080369 N, 37.534226 E and its radius was 0 km. In this most likely high risk period, 4,838 observed cases occurred during a 4-month period when only 1,830.9 cases were expected to occur. Four secondary high risk periods occurred from November 2013 to February 2014 in Debaytilatgen and Gozamin districts, from December 2014 to June 2015 in Dejen and Shebel Berenta, from July 2014 to October 2014 in Debubachefer/Dangila, FagitaLekoma/Semenachefer/Mecha and BahirDar Zuriya districts, and from November to February 2014 in Anikasha Gwagusa/BanjaShekudad/Wenberma and Zegem districts (Table 1).

**Table 1:** Results from space-time permutation scan statistical analysis of childhood diarrhea in northwest Ethiopia, from July 2013 to June 2015.

Cluster	Districts	Location	Start date	End date	Obs^a^	Exp^b^	GLR	p-value
Primary *	Huletej Enese	11.080369 N, 37.534226 E/0 km	2014/3	2014/6	4838	1830.9	1714.9	< 0.001
Secondary	Debaytilatgen/Gozamin	10.300307 N, 37.593736 E/2.8 km	2013/11	2014/2	6650	3827.3	869.7	< 0.001
Secondary	Dejen/Shebel Berenta	10.095555 N, 38.090118 E / 18.84 km	2014/11	2015/6	6719	4891.8	313.1	< 0.001
Secondary	Debubachefer/Dangila/FagitaLekoma/Semenachefer/Mecha/BahirDar Zuriya	11.415083 N, 36.563264 E/ 72.56 km	2014/7	2014/10	6235	5273.1	85.02	< 0.001
Secondary	AnikashaGwagusa/BanjaShekudad/Wenberma/ Zegem	10.510300 N, 36.533224 E/ 18.36 km	2013/11	2014/2	3460	2773.3	79.8	< 0.001

*Most likely cluster

Obs^a^ = observed cases, Exp^b^ = expected cases, GLR = Generalized likelihood ratio

### Pattern of climate variability

Two years of monthly averages of climatic variables of the study sites revealed that the monthly average mean temperature (±SD) was 18.0°C (±3.6 ^o^C) with a range of 8.5°C occurred in main rainy season to 27.8°C in dry season, whereas the mean monthly rainfall (±SD) was 158.3 mm (±193.7mm) with a range of 0 in dry season to 1,042.6 mm in main rainy season. The mean relative humidity (±SD) was 61% (±22%), with maximum of 99.0% in main rainy season and a minimum of 20.0% in dry season.

**Fig 5:**
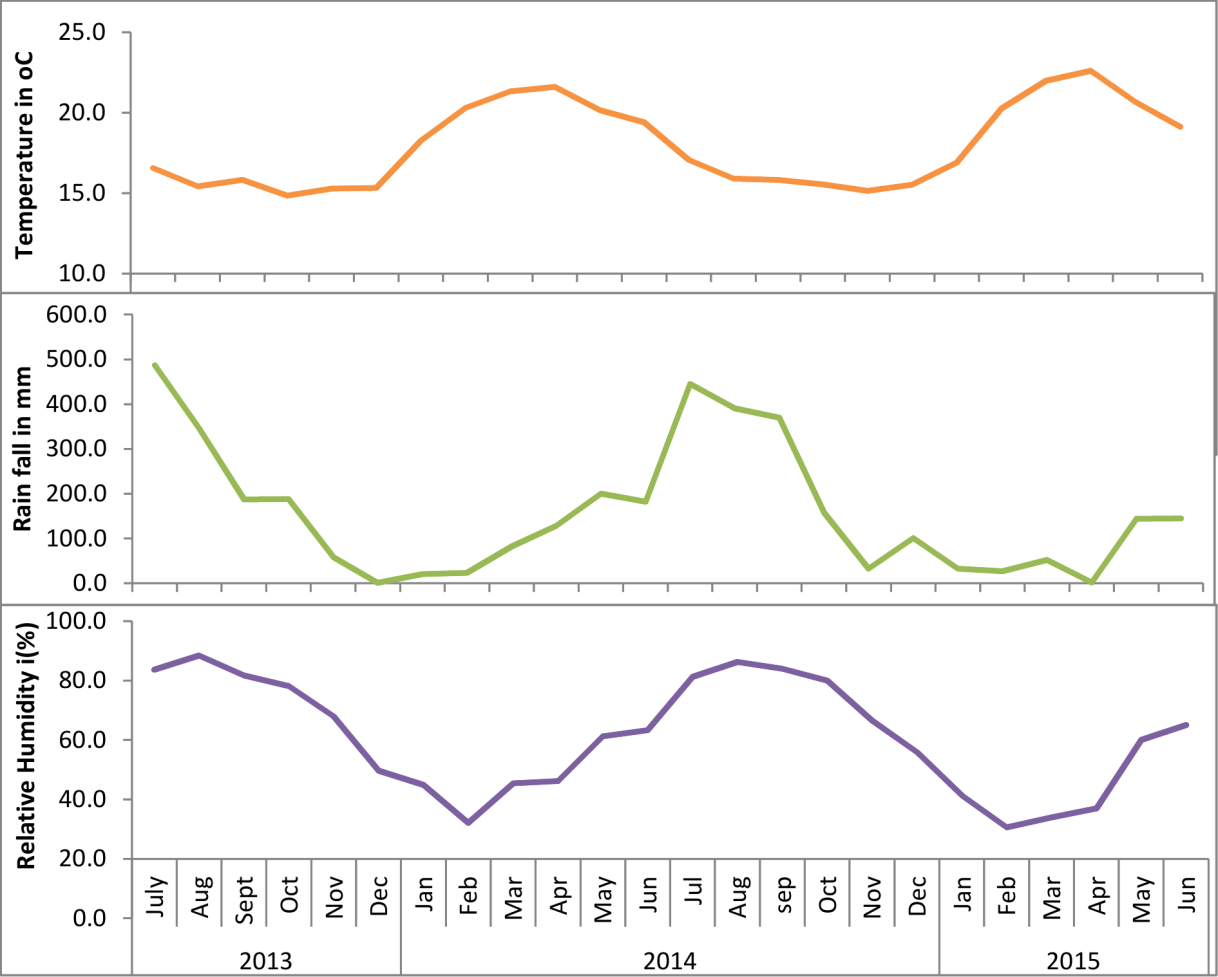
Trends of monthly average temperature, rainfall and relative humidity in northwest Ethiopia, from July 2013 to June 2015.

The pattern of monthly average temperature in this 24 month period revealed that temperature above the monthly average mean temperature (18.0°C) was observed in the period between February and June in dry, short and main rainy season, whereas below the monthly average mean temperature (18.0°C) was observed in the period between July and January during main rainy season and dry season. The highest monthly average temperature was observed in April during short rainy season (21.6°C in 2014 and 22.6°C in 2015) whereas the lowest monthly average temperature was observed in October during dry season (14.8°C in 2013 and 15.5°C in 2014) (Fig 5).

Rainfall above the monthly average mean rainfall (158.3 mm) was observed in the period between July (main rainy season) and October (dry season) in 2013 and between May (short rainy season) and October (dry season) in 2014 whereas below the monthly average mean rainfall (158.3 mm) was observed in the period between November 2013 (dry season) and April 2014 (short rainy season) and between October 2014 (dry season) and April 2015 (short rainy season). The highest monthly average rainfall was observed in July (486.4 mm in 2013 and 445.4 mm in 2014) during main rainy season whereas the lowest monthly average rainfall was observed in November (1.06 mm in 2014) and February (26.7 mm 2015) during dry season (Fig 5).

Humidity below the monthly average mean relative humidity (61%) was observed in the period between December and May whereas above the mean relative humidity (61%) was observed in the period between June and November. The lowest monthly average relative humidity was observed in February (32% in 2014 and 31% in 2015) whereas the highest monthly average relative humidity was observed in August (88% in 2013 and 86% in 2014) (Fig 5).

## Effect of climate variability on childhood diarrhea

### Spearman's correlation analysis

Correlation analysis was conducted to quantify the relationship between monthly morbidity of diarrhea and climatic variables during the study period, with a lag of 0, 1 and 2 months. Monthly average temperature and number of monthly childhood diarrhea cases showed positive correlation at lags of 0, 1, and 2 months. The correlation decreased as the number of lags increased from 0 to 2 months and the positive correlation of temperature with the number of childhood diarrhea cases was significant. Monthly mean rainfall and number of monthly childhood diarrhea cases showed negative correlation at all lags (0, 1, and 2 months). All lag-months were significant and the correlation decreased as the number of lags increased. Monthly mean relative humidity was inversely correlated with the number of childhood diarrhea cases (ranged from -0.33 to -0.37) (Table 2).

**Table 2:** Correlation between monthly childhood diarrhea cases and mean monthly climatic variables in northwest Ethiopia, from July 2013 to June 2015.

Monthly mean climate variables	Lag-months	Spearman's r	p-value
Temperature (^o^C)	0 months	0.37	<0.001
	1 month	0.30	<0.001
	2 months	0.17	<0.001
Rainfall (mm)	0 months	-0.19	<0.001
	1 month	-0.28	<0.001
	2 months	-0.31	<0.001
Relative humidity (%)	0 months	-0.33	<0.001
	1 month	-0.37	<0.001
	2 months	-0.34	<0.001

### Regression analysis

There was collinearity among climatic variables for lag 0 to lag 2 months. Climate variables without lag were entered for multivariate negative binomial regression analysis since the VIF between explanatory variables was lower than 4 (average monthly temperature (^o^C), average monthly rainfall (mm) and relative humidity (%) had 1.89, 2.12 and 3.23 variance inflation factors respectively). In the crude correlation relationship, a positive association was found between monthly average temperature and childhood diarrhea whereas rainfall and relative humidity were inversely correlated with the number of childhood diarrhea cases. Multivariable regression results show that monthly average temperature, monthly average rainfall and relative humidity were statistically significantly associated with the rate of childhood diarrhea. There was a significant positive association between monthly average temperature (IRR = 1.019; 95%CI 1.0034, 1.0347) and the rate of childhood diarrhea and between monthly average rainfall (IRR = 1.0004; 95%CI 1.0001, 1.0007) and the rate of childhood diarrhea. There was a significant inverse relationship between relative humidity and rate of childhood diarrhea (IRR = 0. 3915; 95%CI 0.2721–0.5631) (Table 3).

**Table 3:** Negative binomial regression analysis of the effect of climate variability on childhood diarrhea in northwest Ethiopia, from July 2013 to June 2015.

Variables	Crude IRR (95% CI)	Adjusted IRR^a^ (95% CI)
Monthly average temperature (^O^C)	1.046 (1.035–1.058)***	1.019 (1.0034, 1.0347)*
Monthly average rainfall (mm)	0.9995 (0.9993–0.9997)***	1.0004 (1.0001, 1.0007)**
Monthly average relative humidity (%)	0.405 (0.332–0.494)***	0.3915(0.2721, 0.5631)***

*p-value <0.05

**p = 0.01

***p-value <0.001

IRR = Incidence Rate Ratio, 95% Confidence Interval

^a^Adjusted IRR 95%CI indicates all the climate variables entered in the final model to investigate the independent effect of each climate variable by control the confounding effect between explanatory variables and outcome variable

## Discussion

The results of this study indicate that almost all high risk periods were located in districts of the East Gojam Zone and begin in the dry season. A relatively high incidence rate of childhood diarrhea was observed during the pre-rainy season and peaked incidence rate was observed at the beginning of rainy season, in June. The highest incidence rate of childhood diarrhea in the study area is attributed to the limitations or lack of sanitation facilities, hygienic practice, inadequate safe drinking water [50], the low nutritional status of children [51] and increased transmission by flies as they bred intensively during pre-rainy season [52]. A cross sectional study conducted in 2015 found that nearly 70% of the households (43.0% had unimproved sanitation and 25% used open defecation) in East Gojjam zone districts had no adequate sanitation [50].

The incidence of childhood diarrhea was higher among males than females in this study. This finding is consistent with other findings reported in the literature [53]. The difference in incidence between males and females under five years of age may be because boys are more active than girls, and thus would have more opportunities to be exposed to environments containing bacteria.

Even though two years data of childhood diarrhea used in the study is not large enough to observe the seasonal patterns of childhood diarrhea in children under five years of age the average incidence with 95%CI of each season and time series graph indicated the presence of seasonal pattern of childhood diarrhea. Specifically, a significant high incidence of diarrhea was observed in pre-rainy season (March to May) compared to other seasons dry (from October to February) and main rainy season (June to September). The pre-rainy seasons exhibit high temperature in the study area (for example the highest monthly average temperature was observed in April (21.6°C in 2014 and 22.6°C in 2015). Studies conducted in different regions revealed that common causes of infectious diarrhea (bacteria) follow seasonal patterns and multiply rapidly in the warmer months, when higher temperatures for longer periods can result in higher than expected diarrhea incidences [9–11]. Further study to confirm and observe the seasonal pattern of childhood diarrhea in the study area using long period datasets is recommended.

The space-time permutation scan statistics provided further evidence for a higher than expected number of childhood diarrhea cases began in dry season as high risk period. The most likely high risk period began in dry season from March to June and three out of four secondary high risk periods also began in dry season. The finding of the study is supported with previous study which showed that seasonal variation of childhood diarrhea, which peaked from January to March and April to June, was observed [54]. Since the importance of high risk periods analysis in epidemiology is to detect irregularities of disease and to minimize morbidity and mortality through timely implementation of disease prevention and control measures [41].

The most likely high risk and two out four secondary high risk periods identified in districts of East Gojjam zone which is supported with previous study, significant number of observed cases of diarrhea in spatial only and in both spatial and temporal identified in districts of East Gojjam zone [54]. A study conducted in the study area revealed that low coverage of improved water supply and latrine facilities were reported in East Gojjam zone districts than other districts [50]. Another study conducted elsewhere showed that the number of diarrhea cases also increased with higher temperature, particularly in those individuals at a lower socio-economic and sanitation status [10].

There are studies indicating that future climate change could exacerbate a number of current health problems including diarrhea diseases [7, 8]. A recent systematic review and meta-analysis of ambient temperature and diarrheal diseases in low, middle and high income countries found that changes in temperature due to global climate change can increase diarrheal disease incidence [11]. Studies on the increased risk of diarrhea as a result of changing temperatures have been documented at a global scale as well [55–57]. A recent study indicated that the projected end-of-century temperature increases of up to 4°C over tropical and subtropical continental areas are associated with mean projected increases of relative risk of diarrhea: 8–11% (± 3–5%) by 2039 and 22–29% (± 9–12%) in the period 2070–2099 [56]. Regression results in this study also confirmed that the average monthly temperature was positively associated with the rate of childhood diarrhea, which is consistent with other studies in the literature [7, 8, 10, 13, 58]. The first reason for excess risk of childhood diarrhea associated with high temperature could be due to rapid multiplication and survival of common causative agents of diarrhea for longer periods, which occur in the warmer months [59]. The second reason may be due to temporal changes in human behavior during hot weather conditions, such as higher water consumption, use of unimproved drinking water sources and less hygiene practice due to scarcity of water since most of improved drinking water sources are out of commission during dry seasons [16].

Waterborne diseases are expected to rise with increases in extreme rainfall and deterioration in water quality following wider drought events [17]. Excessive or heavy rainfall events can mobilize pathogens in the environment and increase surface run-off from fields, transporting them into rivers, springs and wells [15] and such events may also increase the risk of flooding in many areas, increasing human exposure to waterborne pathogens [14]. Drinking water source in Ethiopia are springs and shallow wells that are easily flooded and contaminated by micro-organisms [60]. This study is consistent with findings of previous studies in the literature [7, 8, 10, 13, 58, 61], which demonstrated a positive relationship between rainfall and diarrhea morbidity. The main reason of the positive association is the contamination of drinking water sources as a result of increased surface runoff during heavy rainfall events.

Previous studies have found that relative humidity is directly [62] or inversely related to diarrhea [62–64]. A negative relationship between relative humidity and the risk of diarrhea was observed in this study. There are contradicting arguments as to why relative humidity may or may not directly affect the number of cases of diarrhea through various pathways. The positive relationship argument is that relative humidity is strongly linked to the number of extreme rainfall events, thus its effect on childhood diarrhea morbidity is dependent on the relation between increased rainfall and diarrhea [7]. The negative relationship argument was based on laboratory evidence, which found that some causative agents of diarrhea, particularly rotavirus can remain viable at low relative humidity on object materials for more than 10 days at room temperature [65, 66]. Thus, further research on this relationship is needed to evaluate the complex relationship between relative humidity and diarrhea incidences.

This study has some limitations. Underreporting of the number of cases of childhood diarrhea is one of the limitations of similar studies using data from passive surveillance systems. The other limitation is that notified dates of diseases were used instead of onset of dates, which could be more suitable to linking childhood diarrhea morbidity with the lagged effects of climatic factors. Two years childhood diarrhea data may not be enough to observe clearly the seasonal pattern of diarrhea occurrences. Weekly data with other potential confounding factors such as age, gender, hygiene behavior, household economic and hygiene status may bring more accurate estimation of the effects than monthly reported values. However, only monthly surveillance data (cumulative counts of diarrhea cases) were available for this study.

Despite these limitations, this study revealed some important potential health impacts of climate change in the study area. The climate change-induced factors such as temperature, rainfall and atmospheric humidity were shown to be significantly associated with childhood diarrhea, the most frequent public health concern in Northwestern Ethiopia. The World Health Organization reported that climate variability will be one of the public health challenges to control infectious diseases in the future, particularly in developing countries. Therefore, such study can provide valuable information for public health planners to take countermeasures against the disease [4]. Climatic factors and extreme weather events are known to have serious consequences for human health and are predicted to increase in frequency as a result of climate change in Sub-Saharan African countries [67].

## Conclusions

This study found that high risk periods and seasonal patterns of childhood diarrhea were observed in the study area. Climate variables were associated with the occurrence of childhood diarrhea. Increased temperature and rainfall were associated with increased rate of childhood diarrhea morbidity, whereas the relative humidity was inversely related with rate of childhood diarrhea. Climate change in Ethiopia is already evident in a number of ways [18, 20, 68], in the context of the projected changes of climate factors this study suggests that climate change will have important implications for human health. This study provides a useful insight into an existing relationship between climatic factors and childhood diarrhea, which helps local health departments to develop appropriate climate-change adaptation and preparedness for diarrhea prevention. Moreover, the results of this study may provide a basis for predicting childhood diarrhea using regional and seasonal forecasts of climatic factors in vulnerable areas. Therefore, childhood diarrhea prevention and control strategy should consider local weather variations to improve prevention and control measures of childhood diarrhea. Further study is recommended using large or multiple full year datasets to develop a childhood diarrhea prediction model and to observe the seasonal pattern of childhood diarrhea.

## Abbreviations

Adjusted IRR: Adjusted Incidence Rate Ratio
°C: Celsius
CDD: Childhood diarrhea
CI: Confidence Interval
HMIS: Health Management Information System
GLR: Poisson generalized likelihood ratio
SD: Standard deviation
WHO: World Health Organization
